# Supplementary material for: Tobacco, nicotine, and cannabis use and exposure in an Australian Indigenous population during pregnancy: A protocol to measure parental and foetal exposure and outcomes
Source: PLoS One. 2024 Sep 6;19(9):e0300406. doi: 10.1371/journal.pone.0300406 (PMC11379133; doi:10.1371/journal.pone.0300406)
Supplement: S1 File — Variables extracted from standard National Perinatal Data Collection report, together with variables of interest for this project (i.e., tobacco, nicotine and cannabis use and exposure). (DOCX) [file pone.0300406.s001.docx]

### Supplementary Table 1. **Variables/factors of interest**

Maternal, birthing and neonatal variables extracted from perinatal medical records

| **Variable** | **Definition, unit** | **Scale of measurement/ Category** | |
| --- | --- | --- | --- |
| Address: Maternal place of residence | Postcode | Nominal | |
| Admission to Special Care Nursery (SCN) | Admission of neonate to Special Care Nursery following birth (to 28 days) | Dichotomous: Yes/No | |
| Age | Maternal age at last birthday | Continuous: years | |
| Alcohol use | Maternal interview, self-report of any alcohol use in this pregnancy | Dichotomous: Yes/No | |
| Anaemia | < 110 g/l haemoglobin in venous blood | Dichotomous: Yes/No | |
| Antepartum haemorrhage (APH) | Any antepartum haemorrhage | Dichotomous: Yes/No | |
| Apgar 1,5,10 min | Neonatal score of 0, 1 or 2 for: heart rate, breathing, colour, muscle tone & reflex irritability. Range 0 -10, higher score indicates stronger condition | Ordinal 0-10  Category: 0-6 poorer; 7-10 stronger condition. | |
| Augmentation | The stimulation of ineffective uterine contractions after the onset of labour, to manage labour dystocia. | Dichotomous: Yes/No | |
| Birthing method | Method of birth Spontaneous vaginal delivery (SVB), Lower Uterine Caesarian Section(LUSCS), forceps, ventouse | Nominal: SVB, LUSCS, forceps, ventouse | |
| Birthing location | Postcode | Nominal | |
| Birthweight | 1^st^ weight of neonate following birth | Continuous | |
| Body mass index (BMI) | Maternal BMI. Ratio between weight (kg) and height (cm) as measured by weight divided by height squared = kg/m^2^ | Continuous and Ordinal: BMI:< 18.5 underweight,;18.5-24.9normal, 25+ overweight | |
| Born Before Arrival (BBA) | Birth that occurs before arrival at hospital | Dichotomous: Yes/No | |
| Cannabis | Combusted/non-combusted with/without tobacco | Nominal, Frequency | |
| Cardiac disease | Health practitioner diagnosis of any maternal cardiac disease | | Dichotomous: Yes/No |
| Cigarette use | Maternal smoking <20 weeks and amount | | Dichotomous: Yes/No  Nominal |
| Diabetes Mellitus | Health practitioner diagnosis of pre-gestational diabetes Type 1, Type 2, medication and/or diet controlled. | | Dichotomous: Yes/No  Nominal |
| Duration of labour | Duration from onset established labour to complete birth of neonate | | Continuous: hours |
| Elevated glucose | Maternal elevated glucose where inadequate identification of pre-gestational diabetes or gestational diabetes status exists | | Dichotomous: Yes/No |
| Episiotomy | Perineal incision to facilitate birth of neonate | | Dichotomous: Yes/No |
| Forceps | Instrumental delivery of the neonate via the vagina | | Dichotomous: Yes/No |
| Gestation at 1^st^ antenatal visit | Time from last menstrual period until attendance at 1^st^ antenatal visit; weeks | | Continuous, completed weeks and days |
| Gestation at 1^st^ ultrasound | Time from last menstrual period until attendance at 1^st^ ultrasound; weeks | | Continuous: completed weeks and days |
| Gestational diabetes (GDM) | Health practitioner diagnosis of elevated glucose that develops during pregnancy. Medication and/or diet controlled | | Dichotomous: Yes/No |
| Gestational length | Time since last menstrual period and birth of neonate | | Continuous: completed weeks and days |
| Gravida | Number of times a woman has been pregnant regardless of whether the pregnancies result in a live birth | | Discrete: number |
| Head circumference | Neonatal head circumference at birth, cm | | Continuous: cm |
| Hypertension (pre-gestational) | Health practitioner diagnosis of pre-gestational hypertension | | Dichotomous: Yes/No |
| Hypertension | Maternal elevated blood pressure | | Dichotomous: Yes/No |
| Indigenous status (mother) | Self- report | | Dichotomous: Yes/No |
| Indigenous status (neonate) | Maternal report | | Dichotomous: Yes/No |
| Induction and indicator | The purposeful stimulation of uterine contractions for the purpose of accomplishing delivery, prior to the natural onset of labour. | | Dichotomous: Yes/No |
| Labour complications | Health practitioner diagnosis of labour complications | | Dichotomous: Yes/No |
| Livebirth | Neonatal outcome following the complete expulsion or extraction from its mother which after separation, shows signs of life | | Dichotomous: Yes/No |
| Lower Uterine Segment Caesarian Section | Operative delivery of the neonate from the uterus via the abdomen | | Dichotomous: Yes/No |
| Meconium-stained liquor | Presence of meconium in liquor | | Dichotomous: Yes/No |
| Membranes complete | Presence of complete membranes | | Dichotomous: Yes/No |
| Neonatal abnormalities | Presence of any neonatal abnormalities | | Dichotomous: Yes/No |
| Neonatal body length | Neonatal body length at birth | | Continuous: cm |
| Nicotine | Nicotine products | | Nominal, frequency |
| Number of antenatal visits | Number of antenatal visits recorded in the perinatal record following birth | | Discrete: number |
| Number of cord vessels | Visual inspection of cord after separation from neonate | | Discrete: number |
| Parity | Number of previous pregnancies resulting in live births or stillbirths, excluding the current pregnancy | | Discrete: number |
| Placenta complete | Presence of complete placenta | | Dichotomous: Yes/No |
| Placental abruption | Placental separation prior to birth of the neonate | | Dichotomous: Yes/No |
| Placental lie | Relationship of the maternal axis to the foetal axis | | Nominal: longitudinal, transverse, oblique |
| Placental previa | Placental lie across the cervical os | | Dichotomous: Yes/No |
| Placental size | Diameter of placenta at cross diagonal points in cm. Result multiplied together to find area | | Continuous:cm^2^ |
| Placental weight | Weight of the placenta following drainage of blood | | Continuous: grams |
| Post-partum haemorrhage | > 500ml blood loss in first 24 hours post birth | | Dichotomous: Yes/No |
| Pre-eclampsia – eclampsia | Hypertension, oedema and proteinuria during pregnancy | | Dichotomous: Yes/No |
| Pregnancy complications | Health practitioner diagnosis of pregnancy complications | | Dichotomous: Yes/No |
| Pregnancy induced hypertension | Health practitioner diagnosis of hypertension that develops during pregnancy | | Dichotomous: Yes/No |
| Premature rupture of membrane | Rupture of membranes < 37 weeks gestation | | Dichotomous: Yes/No |
| Presentation | Presentation part of neonate at birth (presenting at the superior aperture of the maternal pelvis) | | Nominal: cephalic, breech, shoulder |
| Previous adverse obstetric history | Health practitioner diagnosis of adverse obstetric history | | Dichotomous: Yes/No |
| Rubella immune status | Rubella IgG antibody level > 10 IU/ml | | Dichotomous: Yes/No |
| Sex of neonate | Sex of the neonate | | Nominal: Male/Female/unknown |
| Sexually transmitted infection | Health practitioner diagnosis of sexually transmitted infection | | Dichotomous: Yes/No |
| Significant adverse medical history | Health practitioner diagnosis of any significant adverse medical history | | Dichotomous: Yes/No |
| Smoking<20 weeks (and amount) | Maternal smoking | | Dichotomous: Yes/No |
| Smoking >20 weeks (and amount) | Maternal smoking | | Dichotomous: Yes/No |
| Spontaneous vaginal birth (SVB) | Unassisted vaginal birth | | Dichotomous: Yes/No |
| Stillbirth | Neonate with no signs of life following the complete expulsion or extraction from its mother | | Dichotomous: Yes/No |
| Third stage method (active) | Method of delivery of placenta and membranes | | Dichotomous: Yes/No |
| Tobacco | Tobacco products: combusted/non-combusted | | Nominal, Frequency |
| Urinary tract infection | Health practitioner diagnosis of any urinary tract infection | | Dichotomous: Yes/No |
| Vaping | Nicotine vaped products | | Nominal, Frequency |
| Ventouse | Assisted birth using a suction cap applied to the neonate’s head. | | Dichotomous: Yes/No |
